# Improving Faculty Performance in Providing Written Feedback to Learners

**DOI:** 10.1002/aet2.70235

**Published:** 2026-08-03

**Authors:** Kimberly A. Alford, Allison M. Schiller, Leigh A. Patterson

**Affiliations:** ^1^ Department of Emergency Medicine East Carolina University—Brody School of Medicine Greenville North Carolina USA

## Abstract

**Background:**

Faculty acknowledged the importance of completing resident and student assessments yet demonstrated difficulty in consistently performing this task. The investigators aimed to increase the completion of written assessments by faculty in an academic medical center between the years of 2019 and 2024, with success defined as assessments of 100% of medical student shifts and 50% of resident shifts in the emergency department.

**Methods:**

To meet the objective, the investigators established faculty assessment targets in 2020 and implemented annual interventions over 4 years, including incorporation into annual faculty evaluations, use of a tracking dashboard, and integration into the compensation plan. Performance was retrospectively analyzed for the year prior to and the 4 years of interventions.

**Results:**

Faculty completed assessments for 70% of student shifts and 28% of resident shifts in the 2019–20 academic year. Completion rates improved incrementally over the 4 years of successive interventions and reached 99% of student shifts assessed and 76% of resident shifts assessed. Secondarily, the proportion of faculty meeting assessment targets increased over the study period, with 100% completion rising from 16% to 56% and ≥ 50% completion rising from 34% to 81%.

**Conclusions:**

Faculty assessment completion improved substantially after implementation of targets, dashboards, and compensation incentives. These interventions increased both the proportion of learner shifts assessed and the percentage of faculty meeting assessment targets, demonstrating a sustainable strategy to enhance feedback practices in academic medicine.

## 1 Introduction

Emergency Medicine (EM) residency programs are required to provide regular feedback to residents to guide their development and regularly assess resident success in advancing against the Milestones [1]. Providing written documentation of residents' performance in the clinical space is key to their development as emergency physicians and key to a program's success in measuring their progress. Documenting feedback in writing is a perennial challenge for faculty. Many studies on feedback investigate barriers to faculty completing assessments or introduce innovative tools and education to encourage faculty to complete assessments [2]. Accrediting bodies including Canada's Royal College of Physicians (RCP) have not delineated a minimum number of workplace‐based assessments needed by each resident [3] and the ACGME specifies that “faculty should provide feedback frequently throughout the course of each rotation” [1]. Assessments are also of paramount importance for students both in grade determination as well as to meet Liaison Committee on Medical Education (LCME) requirements. LCME guidelines (Standard 9) [4] specifically state that “formal feedback occurs at least at the midpoint” and that a “narrative description of a medical student's performance” must be provided for a required clerkship.

In 2020, residency program leadership and the department chair proposed adopting a target of documenting assessments on 50% of resident shifts to capture an accurate representation of the resident performance. To meet this departmental goal, we used the average number of resident and faculty shifts to calculate a faculty individual target of completing one assessment per shift worked with a resident. To provide the most accurate midpoint feedback and clinical grading for students, clerkship directors need an assessment for every student shift; therefore, faculty were tasked with completing an assessment for every student they supervised.

Over the next 5 years, the department enacted a series of interventions targeted at improving the number of assessments completed. We looked at the impact of these interventions on faculty performance.

## 2 Method

### 2.1 Study Design and Participants

The study met requirements for exemption from IRB review and approval. We describe a series of educational quality improvement interventions implemented between July 2018 and June 2024. The study group included all full‐time faculty who supervised resident or medical student learners in the emergency department at East Carolina University (ECU) Health Medical Center during the study period. ECU Health Medical Center is a level 1 trauma and tertiary care academic institution located in eastern North Carolina and is affiliated with the East Carolina University—Brody School of Medicine (ECU‐BSOM). Our institution has a 3‐year categorical emergency medicine program with 36 residents and a 5‐year combined emergency medicine and internal medicine program with 10 residents. Medical student learners rotating through the department annually include an average of 77 fourth‐year students at ECU‐BSOM during their required emergency medicine rotation and an average of 12 visiting students interested in emergency medicine. At the inception of the project, there were 38 full‐time faculty. The number of full‐time faculty grew to 52 by the completion of our interventions. Resident assessments were initiated by the faculty member while student assessments were initiated by the student through the medical school's learning management software. The clerkship coordinator regularly reminded faculty to complete student assessments.

### 2.2 Resident and Student Assessment Data Collection/Sampling

In the EM residency at ECU Health Medical Center, each resident works between 6 and 10 ED blocks per academic year (AY) and averages 19 shifts per block. Students rotating in the department work 14 shifts. Resident and medical student blocks are 4 weeks in duration. The total number of assessments completed by each individual faculty member on resident and student learners was recorded per block. The data collected from AY 2018–19 and 2019–20 served as a baseline. At ECU Health Medical Center, the medical student learners were removed from the clinical space from March 2020 until July 2020 due to the COVID19 Pandemic; therefore, there was no evaluation data for this period.

### 2.3 Interventions

To increase the number of faculty completed assessments on resident and student learners, successive discrete interventions were implemented over a five‐year period (Table 1). In AY 2019–20, departmental leaders created a new rubric for use in annual evaluations of faculty performance in clinical, teaching, research, and service categories. In the teaching portion of the rubric, performance targets for assessing learners were included. Faculty were required to complete an average of one resident assessment for each shift worked with residents and an assessment for every medical student assigned to a shift with them. Failing to meet the target resulted in a rating below “Good.” The rubric also allowed faculty to demonstrate excellence in teaching by completing more than the required number of resident assessments. Dissemination of the rubric began in the spring of 2020 with annual evaluations, and faculty were reminded monthly via email and faculty meetings. Residency and clerkship staff recorded the number of assessments completed each month. The chair reviewed and incorporated the performance into each faculty member's annual evaluation.

**TABLE 1:** Intervention timeline.

Academic year	Intervention
2019/2020	Chair and Education Vice Chair created faculty targets for completing learner evaluations and disseminated
2020/2021	Targets incorporated into annual evaluation rubric to inform faculty teaching scores
2021/2022	Power BI dashboard created to allow faculty to track their individual progress in completing resident evaluations
2022/2023	Targets incorporated into medical school compensation plan as quality incentive metrics
2023/2024	Medical student tracking added to Power BI dashboard and timely deadlines add to rubric

Responding to faculty feedback about tracking their individual performance throughout the year, a dashboard was created in AY 2021–22 using Microsoft Power BI. Each faculty member was granted access to their own unique dashboard and encouraged to view it regularly. Each dashboard included a graph comparing the minimum number of expected versus completed resident assessments by month. Throughout the year, staff updated the dashboard monthly. The chair and division chiefs were able to view all faculty performance. In AY 2023–24, the medical student assessments were added to the dashboard as a similar graph.

In AY 2022–23, the medical school faculty physician practice adopted a CARTs‐based compensation model. The model included an annual incentive component based on clinical performance targets. The department incorporated educational metrics including the resident and student assessment targets with the clinical metrics included in the incentive compensation. If faculty failed to meet either clinical or educational targets, the incentive compensation was withheld.

### 2.4 Key Outcome Measures

We initially compared the percentage of student and resident shifts assessed by faculty per year from our baseline data to each study intervention period. Secondarily, we looked at individual faculty who met the target of a resident assessment per faculty shift. We reported this as the percentage of faculty meeting 100% of the target, 85% of the target, and 50% of the target. These numbers were calculated for the baseline data and each study intervention period.

## 3 Results

**FIGURE 1:**
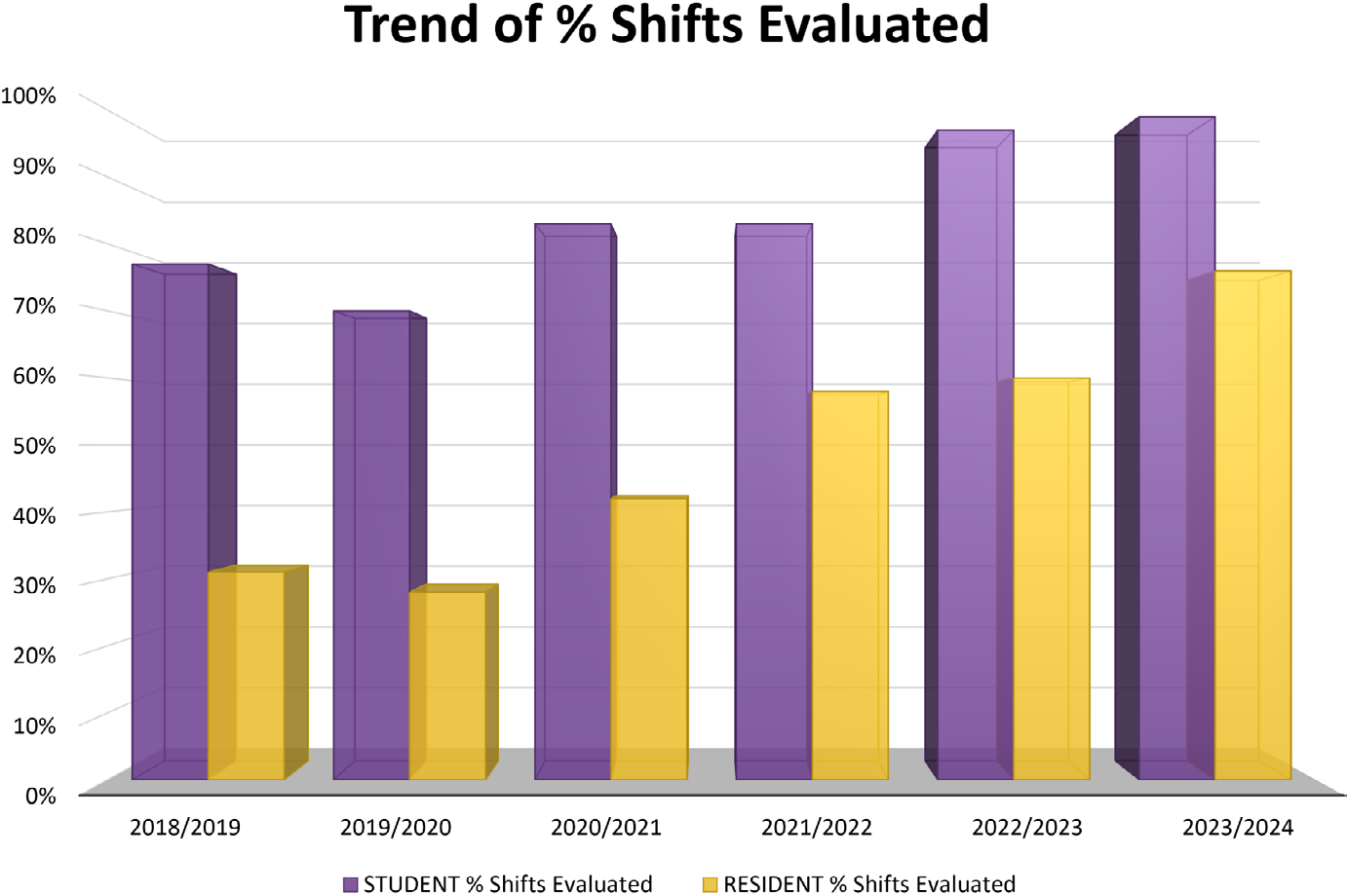
Percentage of both student and resident shifts evaluated per academic year.

The total number of written assessments completed by faculty for both students and residents in each academic year, between 2018 and 2024, is shown in Table 2. The percentage of both student and resident shifts evaluated is shown in Figure 1. The successive interventions culminated in the dramatic increase in resident shifts assessed from 28% to 76% and student shifts assessed from 70% to 99%.

**TABLE 2:** Response to intervention by academic year.

Student assessment data				
Academic year	Annual assessment total	Student shift annual total	Students annual average assessments/Shift	Students % shifts assessed
2018/2019	693	902	0.768	77%
2019/2020	829	1190	0.697	70%
2020/2021	795	960	0.828	83%
2021/2022	968	1160	0.834	83%
2022/2023	975	1010	0.965	97%
2023/2024	1105	1117	0.989	99%

Resident assessment data				
Academic year	Annual assessment total	Resident shift annual total	Resident annual average assessments/Shift	Resident % shifts assessed
2018/2019	2069	6715	0.308	31%
2019/2020	1778	6349	0.280	28%
2020/2021	2748	6617	0.415	42%
2021/2022	3570	6183	0.577	58%
2022/2023	3675	6094	0.603	60%
2023/2024	4757	6281	0.757	76%

Faculty data				
Academic year	Total faculty	% Faculty meeting 100% target	% Faculty meeting 85% target	% Faculty meeting 50% target
2018/2019	38	13%	18%	37%
2019/2020	38	16%	24%	34%
2020/2021	39	21%	26%	44%
2021/2022	40	38%	43%	58%
2022/2023	43	33%	51%	63%
2023/2024	52	56%	69%	81%

**FIGURE 2:**
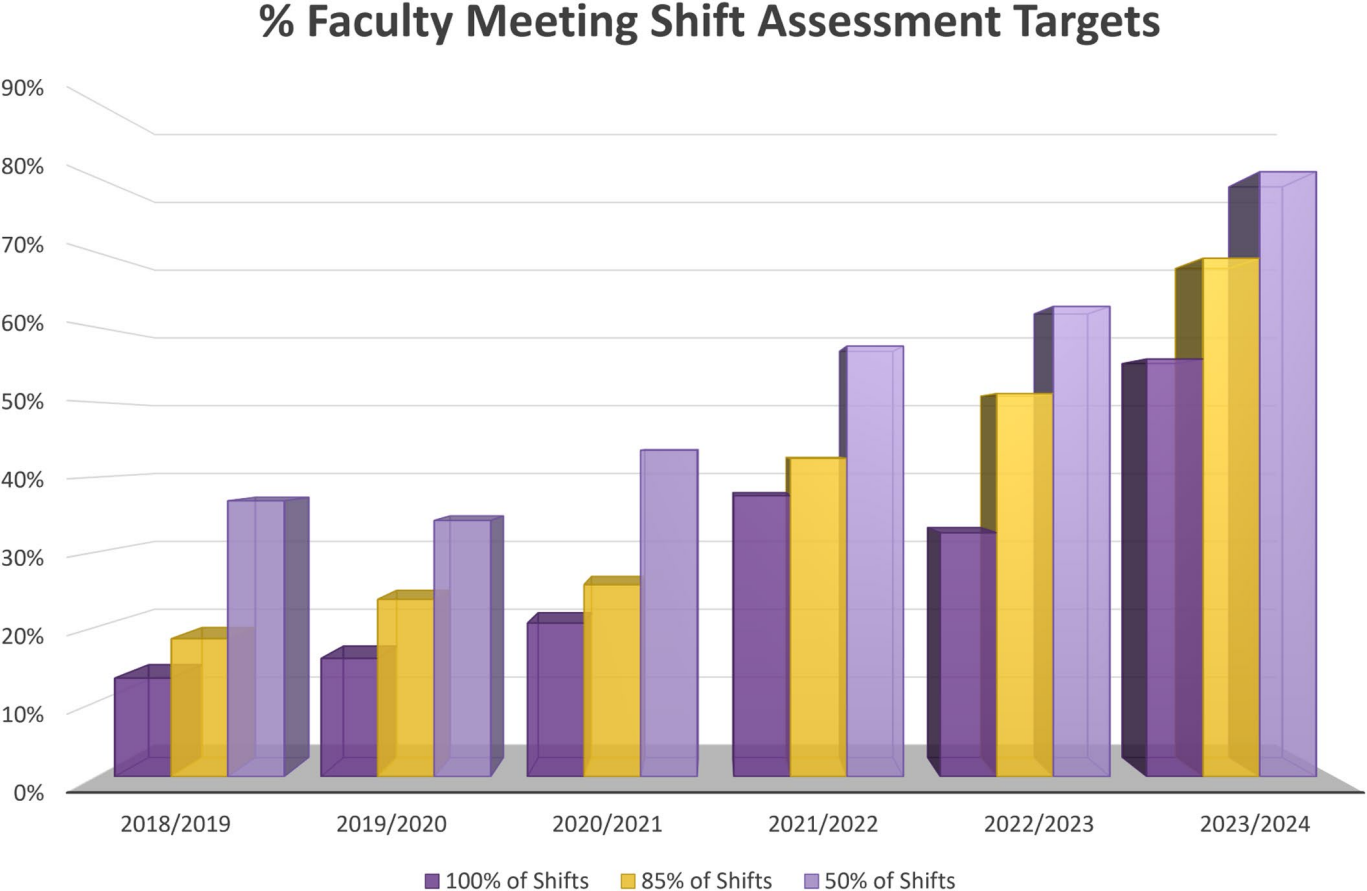
Percentage of faculty meeting 50%, 85%, and 100% of shift assessment targets per academic year.

Our second outcome measure, the percentage of faculty completing 50%, 85%, and 100% of their targeted assessment numbers, is reported in Figure 2. Faculty completing 100% of their targeted assessments increased from 16% to 56%. Faculty completing at least 50% of their targeted assessments increased from about 34% to 81% over the same period.

## 4 Discussion

Previous studies focused on barriers to faculty providing feedback or assessments [2] including fear of harming learners or adding demands to faculty time [5, 6]. Frequently, education on the importance of providing feedback is used to motivate faculty [7]. Other studies introduced a novel assessment tool [8, 9]. In our literature review, we struggled to find studies that measured sustained improvement in faculty performance beyond a year following a single intervention.

We implemented a series of interventions aimed at identifying factors that positively improved faculty performance in documenting feedback as shift assessments. The first intervention was to create targets. Telling faculty they needed to “write more assessments” is too nebulous to yield results. Without a finite target, it is difficult to hold faculty accountable for their performance. By setting a firm goal for resident success (residents receive assessments for 50% of their shifts) and translating this global departmental goal into an individual target for each faculty member, we created a concrete performance baseline. Faculty, especially early‐career faculty, shared that this helped them to better understand expectations of them as teachers.

Faculty also reported they needed help tracking their interval monthly progress toward the annual goal. The improvement following the implementation of a dashboard was markedly higher than simply implementing targets, and the department exceeded the goal of completing an assessment for 50% of resident shifts. We speculate that this is due in part to the visual tool for tracking progress and in part to the effect of observing that the annual evaluation criteria were enforced. As faculty became comfortable using the dashboard, they monitored their personal progress more closely, which ensured more accurate data.

While we saw marginal improvement in the AY 2022–23 in which compensation incentives included educational targets, we saw a larger improvement at the end of the following year after faculty who did not meet targets failed to receive incentive pay. Again, we speculate that this delayed improvement can be attributed to the effect of enforcement.

Our experience with improving assessment of students began with a higher baseline. Two factors contributed to this. First, faculty received links to the necessary form as a request from students through their workplace email accounts which have been demonstrated to improve faculty performance [10, 11, 12]. Second, the clerkship coordinator monitored faculty completion of assessments and regularly reminded faculty personally to complete assessments. Faculty performance and their responses to the interventions mirrored the same success we achieved with resident assessments.

## 5 Limitations

This study was conducted at a single institution. In our academic medical center emergency department, faculty work with residents and students daily. Our results may not be reproducible at a community site in which physician faculty work infrequently with learners. This study focused on improving the overall number of assessments completed but did not address the quality of feedback provided by faculty or the efficacy of a 50% resident shift assessment target. However, residency program directors reported that ACGME resident survey results in the areas of evaluation and teaching improved steadily over the last 3 years. In addition, Clinical Competency Committee members reported a significant increase in the volume of useful performance assessments they now receive.

## 6 Conclusions

The effects seen through our series of interventions demonstrate that several techniques can positively impact faculty teaching performance in documenting assessments of learners. Our interventions used a rubric to define performance benchmarks, visual tools to support regular monitoring of progress to goals, and finally financial incentives for performance. Faculty have shared with us that the scope of the project emphasized to them that teaching performance and specifically assessing learners regularly was a key priority for the department. While each intervention influenced behavior, the cumulative stacking of interventions supported significant changes sustained over 4 years; we believe this is the first paper to report this effect.

## Author Contributions

**Allison M. Schiller:** conceptualization, investigation, writing – original draft, writing – review and editing, data curation, resources. **Kimberly A. Alford:** writing – original draft, conceptualization, investigation, writing – review and editing, data curation, resources. **Leigh A. Patterson:** conceptualization, investigation, writing – original draft, writing – review and editing, data curation, resources, project administration.

## Funding

The authors have nothing to report.

## Conflicts of Interest

The authors declare no conflicts of interest.

## Data Availability

The data that support the findings of this study are available from the corresponding author upon reasonable request.
